# Targeted therapy using engineered extracellular vesicles: principles and strategies for membrane modification

**DOI:** 10.1186/s12951-023-02081-0

**Published:** 2023-09-16

**Authors:** Qisong Liu, Defeng Li, Xiaohua Pan, Yujie Liang

**Affiliations:** 1 National Clinical Research Center for Infectious Diseases, Shenzhen Third People’s Hospital, Southern University of Science and Technology, Shenzhen, China; 2 Department of Orthopaedics, The Second Affiliated Hospital of Shenzhen University (People’s Hospital of Shenzhen Baoan District), China, Shenzhen, 518000 China; 3 Department of Gastroenterology, Shenzhen People’s Hospital (The Second Clinical Medical College, Jinan University, The First Affiliated Hospital, Southern University of Science and Technology, Shenzhen, 518020 China; 4 Department of Child and Adolescent Psychiatry, Shenzhen Kangning Hospital, Shenzhen Institute of Mental Health, Shenzhen Mental Health Center, Shenzhen Clinical Research Center for Mental Disorders, Shenzhen, 518020 Guangdong China

**Keywords:** Exosome, Extracellular vesicles, Surface modification, Targeted delivery

## Abstract

Extracellular vesicles (EVs) are 30–150 nm membrane-bound vesicles naturally secreted by cells and play important roles in intercellular communication by delivering regulatory molecules such as proteins, lipids, nucleic acids and metabolites to recipient cells. As natural nano-carriers, EVs possess desirable properties such as high biocompatibility, biological barrier permeability, low toxicity, and low immunogenicity, making them potential therapeutic delivery vehicles. EVs derived from specific cells have inherent targeting capacity towards specific cell types, which is yet not satisfactory enough for targeted therapy development and needs to be improved. Surface modifications endow EVs with targeting abilities, significantly improving their therapeutic efficiency. Herein, we first briefly introduce the biogenesis, composition, uptake and function of EVs, and review the cargo loading approaches for EVs. Then, we summarize the recent advances in surface engineering strategies of EVs, focusing on the applications of engineered EVs for targeted therapy. Altogether, EVs hold great promise for targeted delivery of various cargos, and targeted modifications show promising effects on multiple diseases.

**Graphical Abstract:**

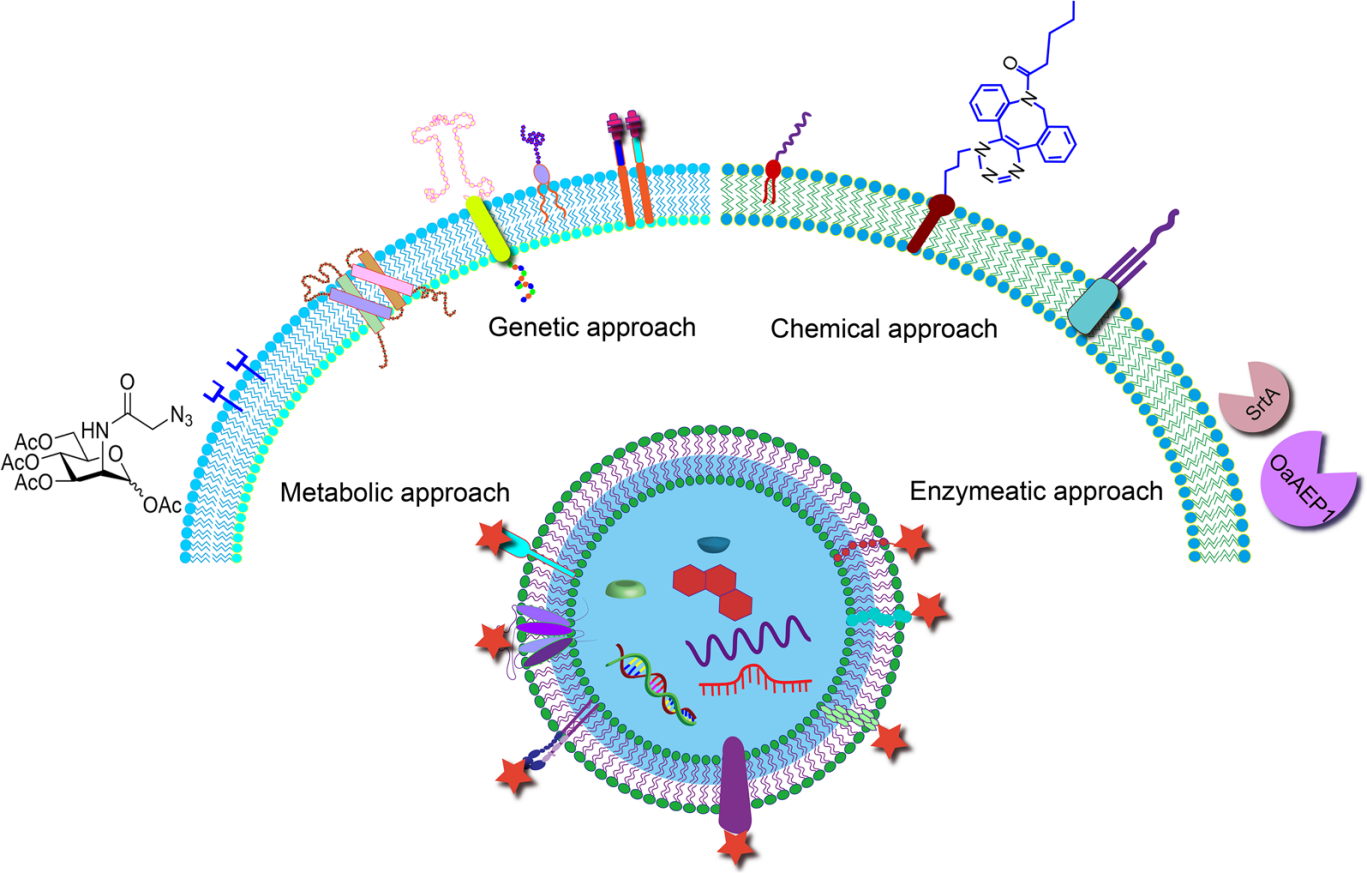

## Background

Extracellular vesicles (EVs) are membrane-bound vesicles secreted by almost all cells. They were first discovered as unwanted waste materials released by cells [1]. Then, EVs gained the spotlight after their role in mediating cell–cell communication was revealed [2]. A PubMed search on “*extracellular vesicles* or *exosomes*”, retrieved 41,475 results by June 30, 2022, most of which were published in the past 10 years. One reason for the extensive attention on EVs is their excellent potential in therapy development.

EVs have therapeutic potential as they could modulate recipient cells by transporting regulatory molecules from parental cells. Mesenchymal stem cell (MSC) therapy showed therapeutic effects on several diseases as they have the immune modulatory capacity and could promote tissue regeneration [3]. EVs derived from MSCs (MSC-EVs) have similar effects as their parental cells and represent alternatives for MSC therapy, as MSC-EVs have less risk of immune rejection and teratoma formation, and a simpler process of production, storage, transportation and administration [4]. Several published or ongoing clinical trials are using MSC-EVs to treat various diseases [5]. Nassar et al*.* conducted a single-center, randomized, placebo-controlled, phase II/III clinical pilot study [6]. The results indicated that EVs from umbilical cord-derived MSCs could ameliorate the progression of chronic kidney diseases as revealed by the significant improved enstimated glomerular filtration rate, serum creatinine level, blood urea and urinary albumin creatinine ratio.

EVs have natural membranes to encapsulate and protect the cargos during circulation, and are excellent delivery vehicles for therapy development [7]. Unlike other delivery vehicles, such as nanoparticles or liposomes, EVs are biocompatible, with a long circulating half-life, minimal or no inherent toxicity issues, and can penetrate biological barriers. They are suitable for delivering nucleic acids, proteins, lipids, and therapeutics. Most importantly, autologous EVs are easily obtained from blood or other body fluids, making them extremely safe without ethical issues, immune rejection, and side effects. Thus, scientific and commercial research on using EVs as delivery vehicles is exploding [8].

However, there are also some limitations in using EVs for delivery. First, EVs per se have regulatory functions, which may counteract the therapeutic purpose. For example, tumor cell-derived EVs may facilitate tumor invasion, so using them as delivery vehicle should be cautious [9, 10]. Second, naturally produced EVs lack targeting specificity. They are largely distributed to liver, lungs, kidneys and spleen after systemic administration [11]. While, the tumor distribution of unmodified tumor cell-derived EVs is minimal as reported by Smyth et al. [12]. So, targeting modification is required to improve efficiency and minimize side effects. To solve this problem, plenty of methods are developed to introduce targeting motifs on EVs surface [13–15]. In this review, we summarize and update recent advances in surface engineering strategies of EVs, focusing on their applications for targeted therapy.

## Biogenesis, composition, uptake, function, and application of EVs

### Biogenesis of EVs

Extracellular vesicles can be classified into exosomes, microvesicles (MVs), and apoptotic bodies based on different sizes and biogenesis pathways, among which the first two are mostly studied for therapy development. MVs are generated directly through the outward budding of plasma membranes, and are launched by specific stimuli, such as inflammation and hypoxia [16–18]. Cytoskeleton remodeling and externalization of phosphatidylserine are the two mechanisms involved in MV formation [19, 20].

**Fig. 1:**
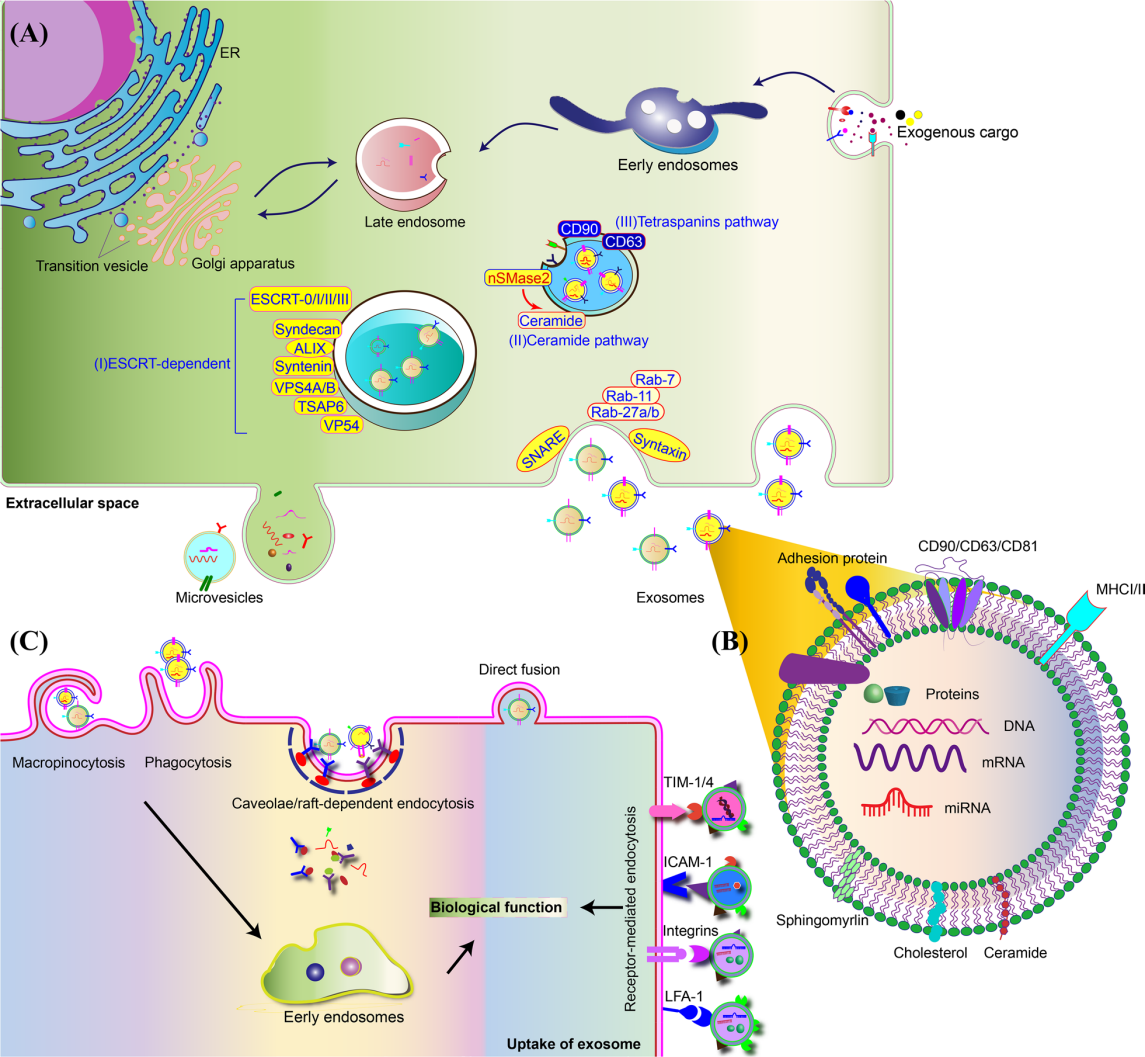
Biogenesis of EVs and the mechanisms involved in the uptake of EVs by the recipient cell. **A** Exosomes are generated as ILVs within MVBs and secreted after MVBs fuse with the plasma membrane. Cargo sorting and ILV formation are regulated by ESCRT-dependent, ceramide and tetraspanin pathways. the trafficking and fusion of MVBs to the plasma membrane are regulated by Rab-7, Rab-11, Rab-27a/b, SNARE, and Syntaxin. Microvesicles are generated directly through the outward budding of plasma membranes. **B** EVs have bilayer lipid membrane, and protein and nuclei acid contents. EVs are composed of lipid membrane and protein, nuclei acid contents. CD63, CD81, and CD9 are the common membrane proteins of EVs. **C** EVs can be internalized by recipient cells through micropinocytosis, phagocytosis, caveolae/raft-dependent endocytosis, direct fusion, and receptor-mediated endocytosis. LFA-1, ICAM-1, CD81 and CD9 on vesicle membranes, are important for the binding and uptake of EVs

Exosomes are generated in a highly sophisticated process within the endocytic system (Fig. 1). During their biogenesis, early endosomes, produced by the invagination of the cell membrane, mature into late endosomes or multivesicular bodies (MVBs) with intraluminal vesicles (ILVs) inside. The ILVs are generated by the inward budding of the endosomal membranes, and exosomes are ILVs secreted extracellularly when MVBs are fused with the plasma membrane [21].

The detailed mechanisms regulating cargo sorting and ILV formation include three pathways, namely the endosomal sorting complex required for transport (ESCRT)-dependent pathway, the ceramide pathway and the tetraspanin pathway (Fig. 1) [22]. The ESCRT-dependent pathway is better characterized than the other two pathways, and involves four protein complexes, ESCRT-0, -I, -II, -III, and more than twenty proteins [23]. During ILV formation, ESCRT-0 and -I recruit cargos and ESCRT-II. Subsequently, ESCRT-II recruits ESCRT-III to promote ILV budding [23]. Other proteins, including syndecan, ALG-2-interacting protein X (ALIX), syntenin, vacuolar protein sorting-associated protein 4A/B (VPS4A/B), tumor-suppressor-activated pathway 6 (TSAP6), and VP54, serve as auxiliary components for the ESCRT machinery in regulating cargo sorting and membrane budding [24]. Ceramide and tetraspanins can promote the ILV formation independent from the ESCRT machinery. Ceramide, a cone-shaped lipid, is enriched in exosomes and can stimulate ILV formation [25]. GW4869, on the other hand, is a nSMase2 inhibitor that impedes exosome production by obstructing ceramide generation. Tetraspanins, such as CD63, CD81, and CD9, are widely distributed in exosomal membranes, which promote ILV by organizing the tetraspanin-enriched microdomains [26]. Besides the ILV formation and cargo sorting, the trafficking and fusion of MVBs to the plasma membrane are regulated by several proteins, including Rab-7, Rab-11, Rab-27a/b, SNARE, and Syntaxin [27].

### Composition of EVs

The size of exosomes ranges from 30 to 150 nm, while MVs have a size range between 100 and 1000 nm. Due to the technical limitations of current purification methods, exosomes or EVs are often isolated together with small MVs, and large MVs can be purified by differential centrifugation [28].

The lipid bilayer membrane of EVs protects the encapsulated molecules, including metabolites, proteins and nucleic acids. Due to different biogenesis pathways, exosomes and MVs have distinct contents and membrane structures. MVs have the same structure and membrane proteins as the plasma membrane and internal contents as the cytosol from parental cells. Exosomes have some inherent features from donor cells but some features are also common among exosomes. The exosomal membrane is composed of many lipids similar to the plasma membrane, but the composition is different from cells, which generate membranes with high rigidity to facilitate the process of internalization [29, 30]. Exosomes from various cell types share some common membrane proteins, including CD63, CD81, and CD9 [31]. Exosomes also have some proteins in common for the internal contents, such as heat shock proteins and ESCRT-associated proteins. Specific proteins or nucleic acids can also be sorted into exosome through ESCRT-dependent or independent pathways (Fig. 1) [32, 33]. Also, the content of exosomes can vary in specific cells upon environment changes, such as hypoxia [34]. Rong et al*.* found that hypoxic pretreatment upregulated the expression of miR-216a-5p in MSC-EVs, which enhanced their therapeutic effect on osteoarthritis [35].

### Uptake of EVs

Various uptake mechanisms of EVs have been reported, including micropinocytosis, phagocytosis, caveolae/raft-dependent endocytosis, receptor-mediated endocytosis, and direct fusion (Fig. 1) [36]. Receptor-mediated endocytosis endows EVs with targeting ability, and supports specific cell–cell communication in vivo, especially long-distance communication [37]. It can occur on lectins, adhesion molecules, and specific receptor-ligand partners. Several lectins, including c-type lectin, galectin 5 and sialic acid on vesicle membranes mediate the endocytosis of EVs by specific cell types [38–40]. Also, adhesion molecules, including integrin, LFA-1, ICAM-1, CD81 and CD9 on vesicle membranes, play essential roles in binding and uptake of EVs [38, 41–43]. Also, several receptor-ligand interactions, such as heparin sulfate proteoglycans-fibronectin, TIM receptors-phosphatidylserine, and epidermal growth factor receptor (EGFR)-epidermal growth factor (EGF), mediate endocytosis of EVs [44–47].

### Biological function of EVs

The major role of EVs is to mediate cell–cell communication by transferring regulative molecules [48]. For example, it’s reported that EVs derived from tumor cells could be specifically internalized by organ-specific cells to prepare the pre-metastatic niche in specific organ, explaining the “seed and soil” hypothesis [43]. Besides, EVs are considered a means to remove excess or unnecessary substances from cells to maintain homeostasis [49]. For example, cancer cell-derived exosomes support the chemo-resistance by actively exporting drugs out of the cells [50].

### Application of EVs

EVs are applicable in disease diagnosis and disease treatment due to the inherited features from their parental cells. First, EVs are emerging as a promising tool for liquid biopsy. As diseased cells release EVs in the body fluids, certain molecule detected in these EVs may reflect the onset and progression of disease [51]. For example, glypican-1 (GPC-1) on cancer cell-derived EVs in serum was identified as a diagnostic index for early-stage pancreatic cancer [52]. Second, EVs secreted by functional cells can be used for disease treatment. For example, EVs derived from MSC and chimeric antigen receptor (CAR)-T cells show similar therapeutic effects as their parental cells [53–55]. Since EVs are safe for in vivo treatment, they have higher potential for clinical transformation than the parental cells.

EVs also serve as excellent vehicles for therapeutic cargo delivery. Their lipid membrane can accommodate and provide a protective barrier for therapeutic agents during circulation. Other advantages of EVs as delivery vehicle include biocompatibility, non-toxicity, ability to penetrate biological barriers, and inherent targeting ability. Not surprisingly, several biotechnology companies are attempting to develop gene therapy for diseases using EV delivered RNAs [56].

## Cargo loading into EVs

The cargo loading can be performed during vesicle production, by transfecting donor cells, and incubating donor cells with the carto, or after collecting vesicles, by electroporation, incubating EVs with the cargo, sonication, freeze–thaw cycling, saponin-assisted incubation, and extrusion [57]. Table 1 summarizes the cargo loading methods for EVs with their advantages and disadvantages. Selecting a proper method tailored for different cargos can greatly improve the loading efficiency.

**Table 1:** Common cargo loading approaches for EVs

	Approaches	Types of cargos	Advantages	Drawbacks	References
Pre-secretory loading	Transfection of donor cells	Nucleic acids, proteins, and peptides	Convenience in loading nucleic acids, stability	Changes in the parental cells due to the alteration of gene expression and the toxicity of the transfection agents	[59–63]
	Incubation of donor cells	Drugs, nanomaterials	Simple procedures	Low loading efficiency, toxicity to parental cells	[64]
Post-secretory loading	Electroporation	Nucleic acids, drugs, proteins, nanomaterials, peptides	High loading efficiency with optimized processes	Risk of vesicles/cargos aggregation	[65]
	Incubation of EVs	Drugs, nanomaterials, nucleic acids, proteins, peptides	Simple procedures, unchanged exosome integrity	Low loading efficiency, limited scope of cargos (can penetrate the membrane)	[66–69]
	Sonication	Drugs, proteins, nanomaterials	High loading efficiency	Membrane integrity damage, risk of vesicle aggregation	[68, 70, 71]
	Freeze–thaw cycling	Proteins, peptides	Simple procedures	Low loading efficiency, risk of vesicle aggregation and vesicle protein degeneration	[72]
	Saponin-assisted permeation	Proteins, peptides, nanomaterials	High loading efficiency	Disruption of the membrane integrity, toxicity	[72]
	Extrusion	Drugs, nanomaterials, nucleic acids, proteins, peptides	High loading efficiency, uniform vesicle size	Membrane integrity damage	[74]

### Transfection

Transfection is frequently employed to load nucleic acids, proteins and peptides into EVs. EVs are natural nanocontainers of nucleic acids and proteins inherited from donor cells. Therefore, desired molecules can be passively packaged into EVs due to their overexpression in donor cells. Lou et al. transfected the miR-122 expression plasmid into adipose tissue-derived MSCs to fabricate miR-122-loaded EVs [58]. The miR-122-encapsulated EVs could sensitize hepatocellular carcinoma cells to chemotherapeutic agents by altering miR-122-target gene expression. Further, intra-tumor injection of these EVs enhanced the antitumor effects of sorafenib on hepatocellular carcinoma xenograft mouse model.

Besides, desired molecules can be actively sorting into EVs by co-transfecting with an exosome-guiding unit. This method is efficient and can enhance the loading yield. Hung et al. transfected the donor cells with recombinant exosomal protein (CD63 or Lamp2b) and MS2 bacteriophage coat protein, capable of binding to the specific MS2 stem-loop RNA secondary structure [62]. The over-expressed RNA, containing the MS2 stem-loop, could be specifically and efficiently sorted to EVs. Similarly, proteins can be introduced into the secreted EVs with a vesicle guiding unit, greatly increasing the loading yield. Ferreira et al. found that KFERQ-containing proteins could be loaded into EVs depending on the lysosome-associated membrane protein 2, isoform A (LAMP2A) [63]. So, exosomal loading of some proteins, e.g. mCherry, could be realized by tagging them with the KFERQ-like sequence.

### Incubation of donor cells

Incubating the donor cells with specific molecules, such as drugs that are internalized, results in EVs, especially MVs, with those molecules as they inherit donor cell contents. Pascucci et al. incorporated paclitaxel (PTX) into MSCs and produced PTX-loaded MVs for cancer treatment [64]. The PTX-loaded MVs demonstrated strong anti-proliferative activity toward pancreatic cancer cells. However, this method may have low loading efficiency as it is non-specific, and is only suitable for drugs that can easily pass the plasma membrane.

### Electroporation

Electroporation uses an electrical pulse to create pores in the bilayer membrane of EVs through which the loading molecules enter the vesicles. This method has excellent translation potential as the loading efficiency is controllable with established procedures. siRNAs are frequently loaded into EVs through electroporation. Alvarez-Erviti et al. first reported siRNA loading into brain-targeting exosomes using electroporation [65].

### Incubation of EVs

Cargos that penetrate the vesicle membrane at a specific temperature, can be loaded into EVs by incubating them with purified EVs. The method is simple and applicable to small molecules, nucleic acids, proteins and nanomaterials [66–69]. The loading efficiency depends on the hydrophobicity of the cargos, enabling the hydrophobic contact between the cargo and the lipid membrane. Besides passive diffusion, some molecules can be taken in by EVs actively. Betzer et al. reported that glucose coated gold nanoparticles could be loaded into EVs through active uptake [69].

### Sonication

Sonication induces deformation of vesicle membrane, enhancing the penetration of cargos. This method is suitable for loading drugs, proteins, peptides, and nanomaterials, but not nucleic acids, which may degrade under sonication [68, 70, 71]. Although sonication is an efficient and simple method for the cargo loading, it is not suitable for scale-up production.

### Freeze–thaw cycling

Multiple freeze–thaw cycles lead to vesicle membrane damage, allowing the diffusion of cargos [72]. The method is simple and suitable for loading of drugs and proteins; however, freeze–thaw cycling has an adverse impact on the activity of proteins, leading to decreased potency.

### Saponin-assisted permeation

Surfactants, such as saponin, could generate pores on the vesicle membrane, allowing the entry of cargos into EVs [72]. This method has high loading efficiency; however, the surfactant has a strong impact on the drug potency. The hemolytic activity of saponin in vivo has been reported [73]. Thus, a thorough purification following cargo loading is needed.

### Extrusion

In this technique, vesicles and cargos are mixed and compressed by the lipid extruder with 100–140 nm pores. The vesicle membrane is damaged during this process, and cargos are encapsulated into vesicles through membrane reassembling, resulting in high loading and uniform-sized vesicles [74]. However, this method greatly alters the membrane structure of vesicles. It has also been reported that vesicles have an altered zeta potential and are cytotoxic after extrusion [75].

## EVs as an excellent drug delivery system

The development of technology enhancing the delivery of drugs to target sites promotes therapeutic activity and minimizes side effects due to off-target accumulation. Several drug delivery systems have been developed, including EVs, liposomes, and nanoparticles. EVs have advantages over artificial delivery systems as they are biocompatible, have long circulating half-life, have minimal or no inherent toxicity issues, penetrate the biological barriers, and can be autologous [76]. Several therapies have been studied by using EVs as delivery vehicles for small molecules, proteins, and RNA. These engineered EVs are promising in treating different diseases, including cancer, brain disease, etc. [58, 77]. Detailed therapeutic strategies are discussed in the following.

### Delivery of small molecules

Small molecules with high potency but poor aqueous solubility are candidates for EV delivery. Kim et al. loaded the hydrophobic anticancer drug PTX by sonication into to macrophage derived EVs modified with the targeting unit aminoethylanisamide (AA). PTX, could be specifically targeted to lung cancer cells, which have the sigma receptor recognized by the “AA” unit. Upon systemic administration, PTX-loaded EV accumulated in the tumor tissue and exerted excellent therapeutic effects [78]. Another attractive point of EVs is their ability to penetrate the systemic barriers, allowing the drugs to target cells beyond the barriers. Many researcher employed EVs to deliver PTX, doxorubicin and curcumin to the brain [79–81]. Tian et al. used RGDyK peptide modified EVs to deliver curcumin to the ischemic brain, which resulted in a strong suppression of inflammation and apoptosis in the lesion [82].

### Delivery of proteins

Protein and peptide drugs have potent and specific bioactivity. However, due to the large molecular weight, and substantial structural fragility, their pharmacokinetic and pharmacodynamic behaviors are unsatisfactory in vivo. Nanocarriers, such as liposomes, were developed to improve bioavailability and minimize side effects through targeted delivery [83, 84]. Several investigators employed EVs for deliver protein drug delivery because of their advantages over other nanocarriers. Functional proteins are delivered by EVs for cancer therapy development. For example, survivin, an anti-apoptotic protein supporting the viability of cancer cells, is an important anti-cancer target [85]. Its dominant-negative mutant, survivin-T34A, can block surviving to induce the apoptosis of cancer cells. Based on this, Aspe et al. fabricated survivin-T34A-encapsulated EVs by transfection, which induced apoptosis of pancreatic cancer cells alone or in combination with Gemcitabine [86]. Also, proteins were delivered by EVs to cross the blood–brain barrier for brain disease treatment. Catalase, a potent antioxidant, was delivered by macrophages-derived EVs by intranasal administration to treat Parkinson’s disease [68]. The engineered EVs showed great neuroprotective effects in vivo disease models.

### Delivery of RNA

RNAs have significant therapeutic potential as they play crucial roles in regulating the expression and activity of target molecules. Recently, the mRNA vaccine to combat the COVID-19 pandemic received approval for clinical use [87]. However, effective delivery vehicles are needed for most RNA-based therapeutics to protect RNAs during circulation and to target cells without side effects.

EVs are natural carriers for biologically active molecules, including various types of RNAs. Some of these RNAs are specifically sorted to EVs and play important roles in cell–cell communication. For example, MSC-EVs carry more than 150 miRNAs and pre-miRNAs with important regulatory roles in recipient cells [88]. RNAs, especially small RNAs, can be efficiently loaded into EVs by various techniques, including genetic transfection and electroporation [62, 65]. Therefore, EVs are ideal delivery vehicles for RNAs combined with other advantages mentioned above.

The discovery of siRNA was a significant advance in biology, as siRNAs can efficiently regulate gene expression [89]. EV-mediated siRNA delivery has shown satisfactory results in several studies. Zhou et al. encapsulated KRAS siRNA in EVs modified with the iRGD peptide (sequence: CRGDKGPDC) to target cancer cells. The engineered EVs exerted strong inhibition of tumor growth in a mouse model [90]. In addition, siRNAs can be delivered to the brain by EVs for the treatment of ischemic stroke [91]. These EVs were decorated with the brain-targeting peptide, RVG, by tagging to Lamp2b, and HMGB1 siRNA was loaded into EVs through electroporation. The intravenously administered engineered EVs decreased the infarct size efficiently [92].

Both endogenous and exogenous miRNAs can be delivered by EVs to exert regulatory effects. MSC-EVs show great therapeutic potential for several diseases due to the cargo miRNAs. For example, several miRNAs, including miR-126, -30b-3P, -145, and -27a-3p, were identified in MSC-EVs for treating severe COVID-19 [93]. Also, exogenous miRNAs were introduced into EVs from various cells to modify target cells for disease treatment. Tao et al. introduced miR-140-5p into MSC-EVs to overcome the side effects of decreasing extracellular matrix production, successfully preventing osteoarthritis in a rat model [94]. In another study, Matsuyama et al. delivered let-7a miRNA to the EGFR-expressing xenograft breast cancer tissue by GE11 peptide-modified EVs. The EVs could efficiently target the tumor tissue after intravenous injection, and inhibit breast cancer development in vivo [95].

mRNAs also possess a remarkable potential for therapy development [96]. However, mRNA delivery is difficult due to its large size [97]. Even so, several studies proved the efficiency of EV-delivered mRNA in disease treatment. Kojima et al. engineered donor cells to produce exosomes with high yield, selectively packaged specific mRNAs, and delivered them into the cytosol of target cells [61]. Using these designer exosomes, the investigators delivered *catalase* mRNA across the blood–brain barrier, and inhibited neuroinflammation in the mouse model of Parkinson’s disease.

### Delivery of other substances

Recombinant adeno-associated viruses (rAAV) are an efficient gene delivery vector tool for gene therapy due to their long-term action, low toxicity, low immunogenicity and broad tissue tropism [98]. Compared to rAAV, vexosomes, rAAV associated exosomes are a better gene delivery platform, as they have minimized toxicity, prolonged circulation time and specific targeting ability. It was reported that vexosomes had better performance in vivo than conventional AAV vectors, as they had higher transduction efficiency than AAV vectors, and were resistant to rapid clearance by neutralizing antibodies [99]. Khan et al. delivered the suicide gene, inducible *caspase 9* (*iCasp9*), by vexosomes, and intratumorally administrated AAV6-iCasp9 vexosomes combined with a pro-drug (AP20187) showed excellent tumor regression effects [100].

CRISPR–Cas9 system is another powerful and promising gene editing technology for gene therapy. Like other functional genetic substances, the CRIPR-Cas9 system requires delivery vehicle to exert its powerful effect in vitro or in vivo [101, 102]. With their multiple advantages, EVs are excellent candidates for serving as CRISPR–Cas9 delivery vesicles [102–105]. Kim et al. reported that CRISPR–Cas9 loaded EVs could efficiently inhibit the targeting gene, *PARP-1*, and enhance the chemosensitivity of cisplatin in ovarian cancer [106]. However, loading efficiency needs to be increased for therapeutic applications due to the large size. Gee et al. loaded the CRISPR–Cas9 system to exosomes utilizing exosome-homing sorting [107]. The Cas9 protein was loaded into exosomes by chemically induced dimerization, and sgRNA was tethered and released into the vesicles by a viral RNA packaging signal and two self-cleaving riboswitches. The efficient gene editing was demonstrated by the permanent genomic exon skipping in a luciferase reporter mouse and mdx mice after the intramuscular injection of CRISPR–Cas9 carrying EVs. Another efficient CRISPR–Cas9 loading method was developed by Osteikoetxea et al. by reversible heterodimerization of Cas9-fusion with EV sorting partners [108]. Efficient loading of approximately 25 Cas9 protein molecules per vesicle and high functional delivery were obtained with 51% gene editing of the target in HEK293 cells.

## Modification of EVs for targeted delivery

**Fig. 2:**
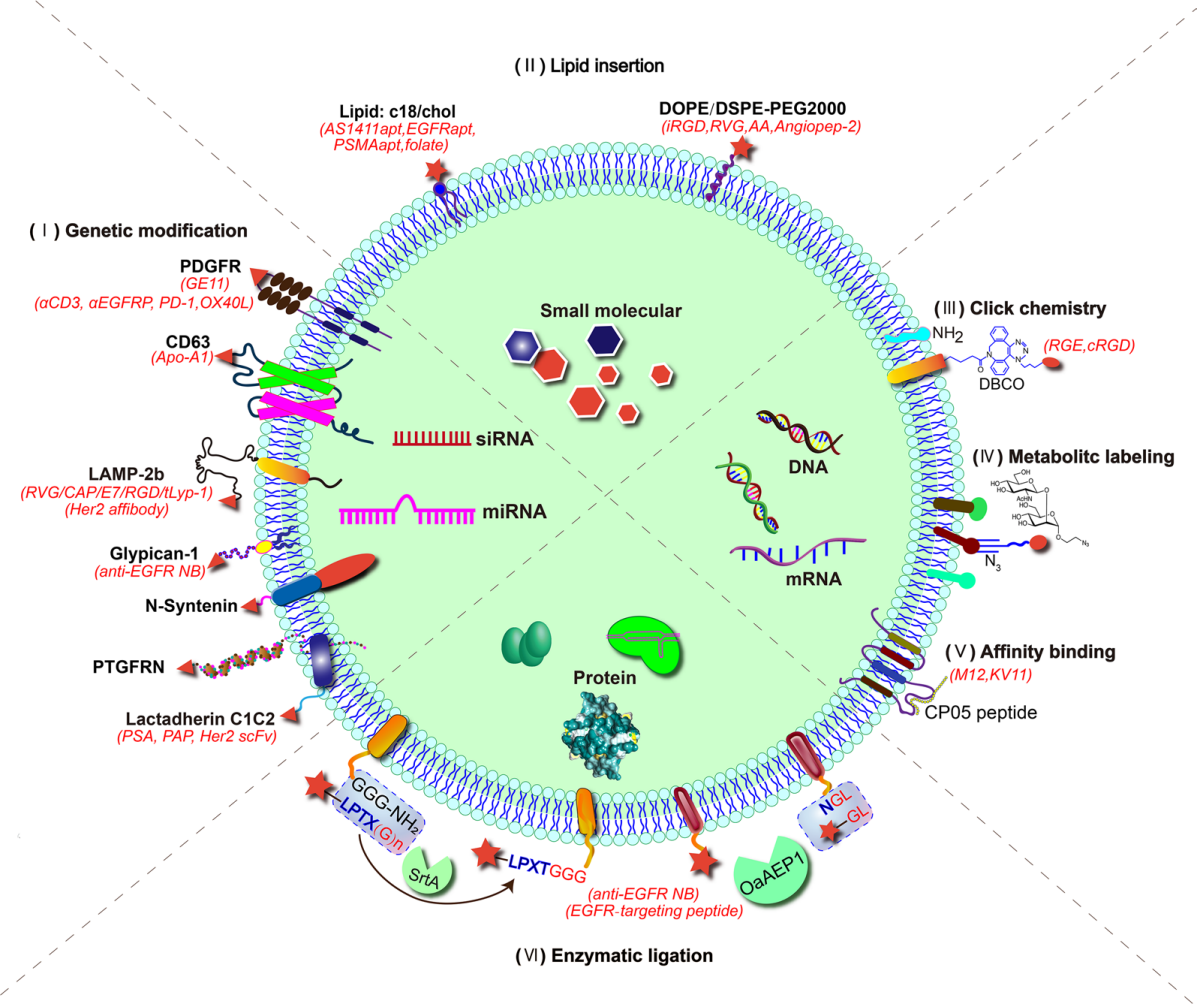
Strategies for the membrane modification and targeted delivery of EVs. Targeting EVs to specific organs or cells can be achieved by membrane proteins and lipids through genetic modification, lipid insertion, covalent ligation, metabolic modification, affinity binding, enzymatic ligation

EVs’ inherent regulative abilities and delivery capacity make them promising candidates for therapy development. Surface modification can further add targeting ability to EVs to enhance the in vivo efficiency and reduce side effects. To date, numerous approaches have been developed to modify the surface of EVs, including genetic modification, lipid insertion, click chemistry, metabolic labeling, affinity binding, and enzymatic ligation (Fig. 2).

### Genetic modification

Genetically engineering donor cells can modify EVs surface with targeting moieties. Generally, in this approach, the targeting peptides or proteins are genetically fused to vesicle membrane proteins or lipid-binding proteins/peptides. Therefore, EVs secreted by these modified cells spontaneously express these targeting moieties on EVs surface (Fig. 2). Examples of using genetic modification to develop therapeutic EVs are listed in Table 2. The genetic modification strategy can be classified into membrane protein-based approach and lipid-protein interaction-based approach.

**Table 2:** Examples of genetic engineering parental cells for surface modification of EVs

Guiding protein	Targeting molecule	Cargo	Effect	References
Lamp2b	RVG peptide	BACE1 siRNA	Delivery of BACE1 siRNA to brain neurons with great potential for Alzheimer’s therapy	[65]
		HMGB1-siRNA	High brain accumulation, effective gene silencing in ischemic strokes	[92]
		Opioid receptor mu siRNA	Delivery of siRNA to the central nervous system and restraining the morphine relapse	[109]
		miR-124	Enhanced gene-drug delivery to the ischemic cortex of the brain and attenuating ischemic damage	[110]
		Nerve growth factor protein and mRNA	Simultaneous delivery of nerve growth factor mRNA and protein to the ischemic cortex	[111]
		mRNA (including nluc, catalase)	Delivery of cargo mRNA to the brain, for rescuing neurotoxicity and neuro-inflammation in Parkinson’s disease	[61]
		circDYM	Amelioration of depression‐like behaviors	[112]
		circSCMH1	Enhanced functional recovery in rodent and nonhuman primate ischemic stroke models	[113]
		Aptamer F5R1 or F5R2	Decreased α-synuclein aggregates in the PD model	[114]
	T7 peptide	Antisense miR-21 (AMO-21)	Delivery of AMO-21 into intracranial GBM	[38]
	CAP peptide	miR-140	Penetration of the dense mesochondrium, inhibits cartilage degradation, and alleviats osteoarthritis progression	[116]
	E7 peptide	Kartogenin	Enhanced chondrogenesis of synovial fluid-derived MSCs (SF-MSCs), and pronounced therapeutic effects in a rat osteoarthritis model	[117]
	RGD peptide	miR-484	Improved vascular normalization, increased ovarian cancer sensitivity towards chemotherapy and prolonged survival time of the tumor-bearing mice after chemotherapy	[118]
	SP94 peptide	Multi-siRNA against GPX4 and DHODH	Enhanced sorafenib-induced ferroptosis and increased hepatocellular carcinoma sensitivity to sorafenib	[119]
	tLyp-1 peptide	Human Sox2 siRNA	Knock-down of Sox2 in cancer cells and reduced stemness of cancer stem cells	[120]
	Her2 affibody	5-Fluorouracil and miR-21	Reversal of drug resistance in colorectal cancer and enhanced efficacy in cancer therapy	[121]
CD63	Apo-A1	miR-26a	Selective binding to HepG2 cells, upregulated miR-26a expression, and decreased cell migration and proliferation	[122]
CD9	ApoB	N.A	Prolonged retention in the brain	[123]
PDGFR	Ge11 peptide	Let-7a miRNA	Specific delivery of let-7a to xenograft cancer cells in RAG2^−/−^ mice	[95]
	scFv antibodies against CD3 and EGFR	N.A	In vitro cytotoxicity towards EGFR-positive triple-negative breast cancer (TBNC) cells in the presence of nonactivated human peripheral blood mononuclear cells, and excellent antitumor activities in a mouse TBNC xenograft model	[125]
	scFv antibodies against CD3 and EGFR, PD-1, OX40L	N.A	Robust anti-cancer immunity, and highly potent inhibition against established TNBC tumors in mice	[126]
Syntenin-1	N.A	Cytokine-binding domains derived from tumor necrosis factor receptor 1 and interleukin-6 signal transducer	Superior efficacy in several inflammatory mouse models	[129]
PTGFRN	N.A	Single-chain version of human interleukin-12	Prolonged tumor retention and great antitumor activity	[130]
CXCR4	CXCR4	TRAIL	Synergistic antitumor effects with carboplatin in the MDA-MB-231Br SCID mouse model	[131]
C1C2 domain of lactadherin	N.A	PSA and PAP	A striking increase in the immune response and improved antitumor efficacy in the tumor models	[133]
	Anti-HER2 scFv antibody	*HChrR6* mRNA	Successful and specific delivery of *HChrR6* mRNA and near-complete growth-arrest of orthotopic BT474 xenografts in vivo	[134]
GPI anchor signal peptides of DAF	Anti-EGFR nanobodies	N.A	Improved EV binding to EGFR-expressing tumor cells	[135]

#### Genetic modification based on membrane proteins

Several membrane proteins are universally expressed on EVs, including CD63, CD81, CD9, Lamp2b, etc. Peptides or proteins genetically fused to the extracellular domain of these proteins can be displayed on the surface. To date, numerous membrane proteins are employed to display targeting moieties on EVs, including Lamp2b, tetraspanins, the transmembrane domain of human platelet-derived growth factor receptor (PDGFR), syntenin-1, prostaglandin F2 receptor negative regulator (PTGFRN), and chemokine receptor 4 (CXCR4).

**Fig. 3:**
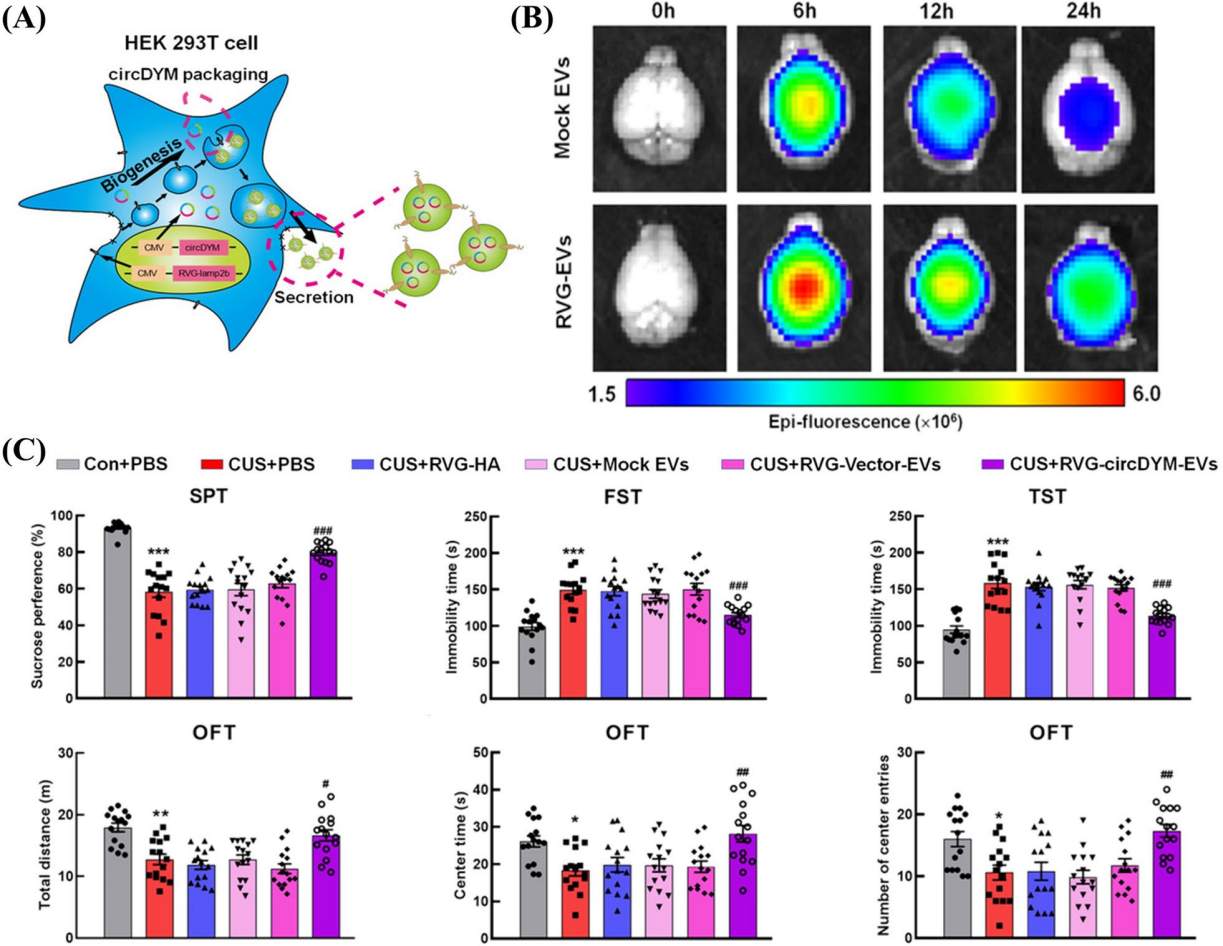
Targeted delivery of circDYM to brain by genetically engineered EVs with RVG peptide for CUS treatment [112]. **A** Schematic diagram of the production of RVG-decorated and circDYM-packaged EVs from HEK 293T cell. **B** Representative near infrared fluorescence (NIRF) images of mice brains after intravenous administration of DiR-labelled mock EVs or RVG-EVs (200 μg) at different time points. **C** The significantly relieved depressive-like behaviors by RVG-EVs delivered CircDYM in CUS mice as measured by the behavior tests, including SPT, FST, TST and OFT (n = 6 for each group) (^*^P, ^**^P, ^***^P vs the Control + RVG-Vector-EVs group; ^#^*P*, ^##^*P*, ^###^*P *vs the CUS + RVG-Vector-EVs group). ^*^P, ^#^*P* < 0.05; ^**^P, ^##^*P* < 0.01; ^***^P, ^###^*P* < 0.001. Two-way ANOVA followed by the Holm–Sidak test were used for the multiple comparisons (Reproduced under the terms of the CC-BY 4.0. Copyright 2022, The Authors, published by Wiley Periodicals, LLC on behalf of the International Society for Extracellular Vesicles)

Lamp2b is the most frequently used guiding protein for decorating exosomes with targeting moieties. It is an exosomal membrane protein and was first utilized by Alvarez-Erviti et al. to equip exosomes with the neuro-targeting peptide, RVG, to deliver *BACE 1* siRNA for treating Alzheimer’s disease [65]. Since then, Lamp2b has been widely applied as an exosome anchoring molecule for targeted therapy development. The Lamp2b-RVG expressing exosomes delivered siRNA, miRNA, mRNA, circRNA, and aptamer to the brain for treating several brain diseases [61, 92, 109–114]. For example, Yu et al. fabricated RVG-decorated EVs (RVG-EVs) by transfecting HEK 293T cells with the plasmid encoding RVG-Lamp2b (Fig. 3) [112]. The modified EVs could deliver circDYM to brain for major depressive disorder (MDD) treatment (Fig. 3A). RVG-EVs showed significantly higher fluorescent intensity in brain than mock EVs as revealed by in vivo imaging system (IVIS) (Fig. 3B). RVG-EVs delivered circDYM (RVG-circDYM-EVs) could significantly alleviate the depressive-like behaviors in the chronic unpredictable stress (CUS) mouse model, indicated by sucrose preference test (SPT), forced swim test (FST), tail suspension test (TST), and open field test (OFT) (Fig. 3C).

Also, Lamp2b was employed to display other targeting peptides on exosomes for therapy development, including T7, CAP, E7, RGD, SP94, and tLyp-1 peptides [115–120]. Besides peptides, affibodies with small sizes but high affinities towards specific proteins can be displayed on exosomes by fusing to Lamp2b. Liang et al. produced Her2 affibody-decorated exosomes to deliver 5-Fluorouracil and miR-21 inhibitor oligonucleotide to Her2-expressing cancer cells [121]. The engineered exosomes could reverse the drug resistance in colorectal cancer and thus enhance the efficacy of cancer therapy.

Tetraspanins, including CD63, CD81 and CD9, are transmembrane proteins abundant on exosomes, which also show potency for the surface modification of exosomes through genetic engineering. Liang et al. fused Apo-A1, the main component of high-density lipoprotein, with the transmembrane domain of CD63 to display it on the external face of the exosomal membrane for the targeted delivery of miR-26a. The engineered exosomes could selectively bind to HepG2 cells, upregulate miR-26a expression, and decrease cell migration and proliferation [122]. The extracellular loops of tetraspanins also allow the incorporation of targeting peptides on the exosome surface. Choi et al. decorated exosomes with ApoB by inserting it between the amino acids 170–171 of CD9, promoting the penetration of blood brain barrier (BBB) through hijacking receptor-mediated transcytosis [123]. The engineered exosomes had prolonged retention in the brain for 24 h after intravenous administration.

The transmembrane domain of PDGFR has been used to display proteins on cell surfaces and to decorate exosomes [124]. Ohno et al. tagged the GE11 peptide on the exosome surface for the targeted delivery of let-7a miRNA to EGFR-expressing breast cancer cells [95]. The intravenously administered exosomes specifically targeted the xenograft breast cancer cells in RAG2−/− mice. Simultaneous binding T-cells and cancer cells presents a new class of cancer immunotherapy by engineered exosomes. Cheng et al. expressed monoclonal antibodies specific to T-cell CD3 and cancer cell-associated EGFR on the surface of exosomes by fusing them with the transmembrane domain of PDGFR [125]. The resulting synthetic multivalent antibodies retargeted exosomes (SMART-Exos) induced the cross-linking of T-cells and breast cancer cells, and showed great antitumor immunity in vitro and in vivo. Later, they displayed the immune checkpoint inhibitors, programmed death-1 (PD-1) and OX40 ligand (OX40L) on the surface of exosomes derived from Expi293F cells together with the two monoclonal antibodies [126]. The resulting genetically engineered multifunctional immune-modulating exosomes (GEMINI-Exos) showed improved therapeutic efficacy compared with the SMART-Exos due to the synergistic effects.

CD63, CD81 and CD9 are established biomarkers for exosomes [31]. However, the heterogeneous presence and abundance of these proteins in exosomes derived from different cell types have been reported [127]. Kugeratski et al. identified syntenin-1 as a universal biomarker candidate protein for exosomes from different cells present at high abundance, as determined by unbiased quantitative proteomic analysis [128]. Therefore, it could also be a universal guiding protein with an increased surface display of exosomes by genetic engineering. Gupta et al. screened several EV-loading protein moieties and found that the N-terminal of syntenin-1 performed best in the joint display of the cytokine-binding domains derived from tumor necrosis factor receptor 1 and interleukin-6 signal transducer [129]. The engineered exosomes showed superior efficacy in several inflammatory mouse models compared to the clinically approved agents targeting the tumor necrosis factor-alpha and interleukin-6 signal pathways.

Besides the above-mentioned guiding membrane proteins, other exosomal membrane proteins may also be utilized for the surface display of exosomes. Prostaglandin F2 receptor negative regulator (PTGFRN) is a membrane protein abundantly expressed on exosomal membranes. Lewis et al. used it to display the single-chain version of human interleukin-12 for cancer therapy [130]. The engineered exosomes showed prolonged tumor retention and profound antitumor activity.

Other than as an anchoring tool, EV membrane proteins could act as targeting motifs. CXCR4 protein plays a critical role in homing MSCs for tumor cell metastasis through the SDF-1/CXCR4 axis. By genetic engineering, Liu et al. overexpressed CXCR4 in MSCs and obtained the CXCR4-rich EVs to deliver TRAIL for treating brain metastases of breast cancer [131]. The engineered EVs showed synergistic antitumor effects with carboplatin in the MDA-MB-231Br SCID mouse model.

#### Genetic modification based on lipid-protein interactions

In addition to membrane proteins, surface modification of EVs can occur on the membrane lipids. In this approach, the targeting moieties are genetically fused to proteins/peptides that can specifically bind to exosomal membrane lipids. Then, the transfected cells can secret modified EVs due to the specific protein/peptide-lipid interaction. This approach is less straightforward than methods based on membrane proteins. Till now, only lactadherin-phosphatidylserine and decay-accelerating factor (DAF)-glycosylphosphatidylinositol (GPI) interaction are employed to decorate EVs for targeting purpose.

Lactadherin is a secretory protein released into the extracellular milieu and specifically binds to the vesicle surface through phosphatidylserine [132]. Thus, the C1C2 domain of lactadherin is employed to display substances on the vesicle surface for therapy. Rountree et al. prepared EVs with two tumor-associated antigens, prostate-specific antigen (PSA) and prostatic acid phosphatase (PAP), by fusing them with the C1C2 domain of lactadherin [133]. The engineered EVs exhibited increased immune response and improved antitumor efficacy in tumor models. Wang et al. also employed the C1C2 domain of lactadherin to display anti-HER2 scFv antibodies for the targeted delivery of *HChrR6* mRNA [134]. The engineered EVs showed great antitumor efficacy as the intravenous administration resulted in near-complete growth arrest in the orthotopic BT474 xenograft model.

Besides Lactadherin, signal peptides derived from DAF could be specifically anchored to GPI for the surface display of EVs. Using this approach, Kooijmans et al. decorated EVs with anti-EGFR nanobodies [135]. The engineered EVs showed significantly improved cell association to the EGFR-expressing tumor cells. Thus, GPI-anchoring represents a novel tool for the surface display of EVs.

### Direct surface modification of EVs

Surface modification can also occur directly on EVs membrane. The membrane is composed of lipids and proteins, allowing modification of lipid insertion, chemical ligation, enzymatic ligation, affinity binding, and metabolic labeling (Fig. 2). In this approach, targeting moieties are introduced to EVs surface through tagging with fragments that can insert into membrane, or react with or bind to membrane molecules, or react with metabolically altered membrane molecules. Table 3 lists several related studies.

**Table 3:** Examples of direct surface modification of EVs

Modification methods	Anchoring motif	Targeting molecule	Cargo	Effect	References
Lipid insertion	DSPE-PEG	RGD	Tetraacetylated *N*-azidoacetyl-d-mannosamine (Ac4ManNAz)	Increased targeting to blood vessels and synergistic therapeutic angiogenesis effect and angiogenesis imaging	[136]
		AA	PTX	Profound ability to accumulate in cancer cells upon systemic administration and improved therapeutic effects	[78]
		Angiopep-2	Docetaxel	Targeted GBM delivery and therapy	[137]
		Angiopep-2	Signal transducers and activators of transcription 3 (STAT3) siRNA	Xenograft growth inhibition due to BBB penetration, and enhanced median survival rate of the tumor-bearing nude mice	[138]
	DOPE-PEG	RVG peptide	N.A	Improved targeting towards the cortex and hippocampus, greatly reduced plaque deposition and Aβ levels, decreased activity of astrocytes, and improved cognitive function of APP/PS1 mice, as determined by the Morris water maze test	[139]
	Cholesterol	AS1411 aptamer	let-7 miRNA	Targeted breast cancer delivery and tumor growth inhibition	[140]
		PSMA aptamer, EGFR aptamer and folate	Survivin siRNA	Tumor growth inhibition in prostate cancer xenograft, orthotopic breast cancer models, and patient derived colorectal cancer xenograft	[141]
	Diacyllipid-PEG	Aptamer sgc8	Doxorubicin	Selective cancer cell targeting and high therapeutic efficacy in vitro	[142]
	C18-PEG, DSPE-PEG, Cholesterol-PEG	AS1411 aptamer	PTX	Increased chemotherapeutic effects and decreased side effects in vivo	[143]
Chemical ligation	Amino group based EDC/NHS coupling chemistry and azide-alkyne cycloaddition chemistry	c(RGDyK) peptide	Curcumin (MSC-derived EVs)	Ischemic brain tissue targeting in the transient middle cerebral artery occlusion mice model, and strong suppression of the inflammatory response and cellular apoptosis in the lesion	[82]
		c(RGDyK) peptide	miR-210	Ischemic brain tissue targeting in the transient middle cerebral artery occlusion mice model and enhanced animal survival rate	[145]
		Neuropilin-1-targeted peptide (RGE)	Superparamagnetic iron oxide nanoparticles and curcumin	BBB penetration, good results for targeted imaging and therapy of glioma in the orthotopic glioma model	[144]
	Thiol group based thiol-maleimide conjugation	Alexa488 (for imaging)	N.A	Quantitative analysis of the cellular delivery and intracellular traffic of EVs	[146]
Metabolic labeling	Azide group originating from L-azidohomoalanine supplement or tetra-acetylated N-azidoacetyl-D-mannosamine in culture medium	Biotin/FITC	N.A	Imaging analysis of EVs in cells and excellent potential for targeted delivery	[147]
	Azide group originating from tetra-acetylated N-azidoacetyl-D-mannosamine in culture medium	Azadibenzylcyclooctyne-fluorescent dyes	N.A	Analysis of the uptake and distribution of EVs in vitro and in vivo	[148]
Affinity binding	CP05	M12 peptide	Phosphorodiamidate morpholino oligomer	enhanced dystrophin-positive myofibers in muscles and functional rescue	[149]
		Thioketal-mPEG (assisted by Chlorin e6 and ultrasound irradiation)	Bmp7 mRNA	Phagocytosis escape from non-target organs, targeted delivery of Bmp7 mRNA into omental adipose tissue (OAT) and induced OAT browning	[151]
		N.A	KV11 peptide	Greatly enhanced anti-angiogenic effects over KV11 peptide	[152]
	Transferrin	Superparamagnetic nanoparticles	Doxorubicin	Excellent in vivo targeting ability and cancer inhibition effect	[153]
	C1C2 domain of lactadherin	EGFR nanobody	N. A	Specific uptake by EGFR-overexpressing tumor cells	[157]
		(RGD)-4C peptide	Neural progenitor cell derived-EVs	Lesion region targeting and suppressed inflammatory response in the MCAO mouse model	[158]
	GPI anchor signal peptide derived from DAF	Anti-EGFR nanobodies	N.A	Improved EV binding to EGFR-expressing tumor cells	[135]
	HepG2 cells derived EVs	Aptamer LZH8	FITC conjugated by DNA hybridization chain reaction	Fluorescence modification of specific EVs	[154]
Enzymatic conjugation	Vesicle membrane protein containing N-terminal glycine and leucine residues	EGFR-targeting peptides or anti-EGFR nanobodies	PTX	Increased accumulation in EGFR-positive cancer cells and significantly increased drug efficiency in a xenografted mouse model of EGFR-positive lung cancer	[156]

#### Lipid insertion

The EV membrane allows hydrophobic insertion of lipids and lipid-tagged molecules, in which the latter is an extensively used surface modification approach for EVs. Targeting peptide or aptamer tagged with a lipid fragment can insert into EVs membrane through a simple mixing and incubation. Compared to other modification approaches, the lipid insertion-based method is simple, inexpensive, rapid, highly efficient, and can be applied to virtually all EV types without perturbing their morphology and biological properties.

The 1,2-Distearoyl-*sn*-glycero-3-phosphoethanolamine-Poly(ethylene glycol) (DSPE-PEG) module, approved by FDA for medical application, is extensively used for anchoring targeting molecules on the surface of EVs, in which the DSPE fragment supports the hydrophobic insertion and PEG fragment provides the stealth effect to reduce protein adsorption on EVs. Wang et al. decorated EVs with RGD peptide through DSPE-PEG, and the modified EVs showed increased targeting towards the blood vessels [136]. Kim et al. also tagged AA to EVs through DSPE-PEG insertion, which could selectively deliver PTX to lung cancer cells and exhibited improved anticancer effects [78]. The targeted delivery of drugs for treating glioblastoma (GBM) requires overcoming the BBB, which was challenging before the application of EVs for delivery. Several studies reported that the DSPE-PEG application supported EV modification for targeting GBM. Wu et al. decorated the exosome-mimetics with angiopep-2 by DSPE-PEG insertion [137]. The modified vesicles could deliver docetaxel to GBM, significantly inhibiting GBM growth with reduced chemotherapy side effects. Liang et al. employed meleimide (Mal)-terminated lipid (DSPE-PEG-Mal) for adding angiopep-2 on the EV surface by lipid insertion and thiol-maleimide conjugation [138]. The modified EVs delivered the signal transducers and activators of transcription 3 (STAT3) siRNA for GBM treatment, which significantly inhibited the growth of orthotopic U87MG xenografts and enhanced the median survival time of the tumor-bearing nude mice.

Similar to DSPE-PEG, 1,2-Dioleoyl-*sn*-glycero-3-phosphoethanolamine-Poly(ethylene glycol) (DOPE-PEG) can also support the surface modification of EVs through lipid insertion. Cui et al. modified MSC-derived EVs with the RVG peptide to treat Alzheimer’s disease [139]. Modified EVs exhibited improved targeting towards the cortex and hippocampus after systemic administration. The RVG-modified EVs greatly reduced plaque deposition and Aβ levels, and the activity of astrocytes. Also, the cognitive function of APP/PS1 mice was greatly improved, as judged by the Morris water maze test.

Cholesterol is another lipid tail often used for modifying the EV surface. EV membrane is enriched in cholesterol, supporting the hydrophobic insertion. Wang et al. decorated EVs with AS1411 aptamer for targeting, which could bind to the highly expressed membrane protein nucleolin on breast cancer cells [140]. The delivery of let-7 miRNA by these engineered EVs showed specific targeting to tumor tissues and an inhibitory effect on tumor growth. Pi et al. found that introducing the cholesterol tag at the tail of the arrow-shaped RNA resulted in the surface display of the RNA aptamer or folate on EVs [141]. Also, the effect of survivin siRNA-loaded EVs was confirmed in prostate cancer xenografts, orthotopic breast cancer models, and patient-derived colorectal cancer xenografts, with PSMA aptamer, EGFR aptamer, and folate-surface modification, respectively.

Besides DSPE and cholesterol, other lipid tags are also employed for the surface modification of EVs. Zou et al. employed diacyl lipid-tagged sgc8 aptamer to modify EVs for doxorubicin delivery, resulting in selective cancer cell targeting and high therapeutic efficacy in vitro [142]. Wan et al. compared the labeling efficiency of lipids, including C18-PEG, DSPE-PEG, and cholesterol-PEG, and found the highest loading efficiency with cholesterol-PEG [143]. Subsequently, they decorated the nanovesicles with AS1411 aptamer through cholesterol insertion and delivered PTX for cancer treatment. The engineered nanovesicles showed increased chemotherapeutic effects and decreased side effects in vivo.

#### Chemical ligation

Chemical ligation approaches are developed based on reactive groups from vesicle membrane lipid or proteins, including amino, carboxy and thiol groups. These groups can biorthogonally react with reactive fragment-tagged peptides. Then the targeting peptides are decorated on EVs surface. The method is robust compared with lipid insertion and affinity binding methods. However, it’s non-specific, and can block some protein–protein interactions and alter the properties of EVs.

**Fig. 4:**
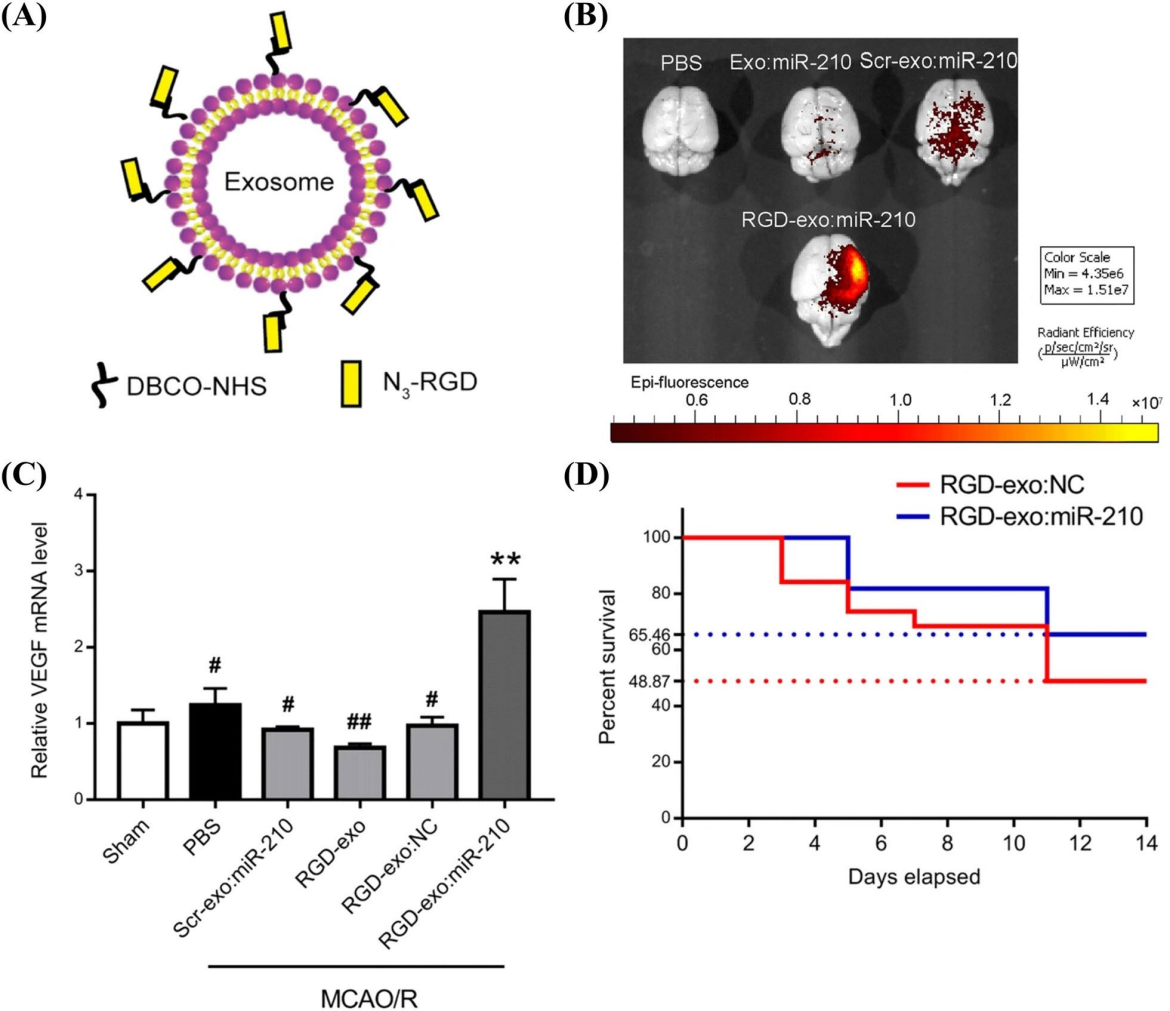
Targeted delivery of miR-210 by chemically modified EVs for MCAO treatment [145]. **A** Schematic diagram of RGD modification on EV surface by EDC/NHS coupling chemistry and click chemistry. **B** NIRF imaging of the MCAO mouse brains 6 h after the intravenous injection of PBS, unmodified EVs with miR-210 (Exo:miR-210), scramble peptide-modified EVs with miR-210 (Scr-exo:miR-210), and RGDyK-modified EVs with miR-210 (RGD-exo:miR-210) (EVs labeled with Cy5.5). **C** The mRNA level of VEGF in the lesion region of MCAO mice 24 h after the intravenous injection of PBS, Scr-exo:miR-210, RGD modified EVs (RGD-exo), RGD modified EVs with controlled RNA (RGD-exo:NC), and RGD-exo:miR-210 (^**^P < 0.01 vs the Sham group; ^#^*P* < 0.05, ^##^*P* < 0.01 vs RGD-exo:miR-210 group using one-way ANOVA followed by Tukey’s post hoc test). **D** Survival rate of MCAO mice after the intravenous administration of RGD-exo-NC and RGD-exo:miR-210 (Reproduced under the terms of the CC-BY 4.0. Copyright 2019, The Authors, published by BioMed Central on behalf of the Journal of Nanobiotechnology)

Amino groups are abundant on vesicle membranes, such as terminal and side chains of membrane proteins, or the membrane lipid, phosphatidylethanolamine. The combination of EDC/NHS coupling chemistry and azide-alkyne cycloaddition chemistry (click chemistry) on amino groups is the most frequently used method for the surface modification of EVs. Jia et al. employed this method to modify EVs with the neuropilin-1-targeting peptide, RGE [144]. The modified EVs delivered the superparamagnetic iron oxide nanoparticles (SPION) and curcumin to the brain for treating glioma. The nanoparticles showed excellent BBB penetration capability and, combined with the SPION-mediated magnetic flow hyperthermia, a potent synergistic antitumor effect. Tian et al. also used this method to modify curcumin-loaded MSC-EVs with c(RGDyK) peptide [82]. The engineered EVs could target the lesion and strongly inhibit the inflammatory response and cell apoptosis in the transient middle cerebral artery occlusion (MCAO) mouse model. Later, the same group applied the modified EVs to deliver miR-210 for treating cerebral ischemia (Fig. 4) [145]. EV modification started with the coupling reaction between NH_2_ group on EV surface and DBCO-NHS (Dibenzocyclooctyne-*N*-hydroxysuccinimidyl ester). Then, RGDyK peptides were introduced to EV surface by click chemistry between N_3_-RGDyK and DBCO-modified EVs (Fig. 4A). In the MCAO mouse model, RGD-decorated EVs showed higher enrichment in the lesion region than unmodified EVs 6 h after intravenous administration under NIRF imaging (Fig. 4B). Further in vivo experiments showed that miR-210 delivered by RGDyK-decorated EVs could promote angiogenesis and enhance the survival of MCAO mice (Fig. 4C and D).

Besides amino group, thiol group also constitutes a reactive site for the surface modification of EVs, interacting with maleimide-tagged molecules by thiol-maleimide conjugation. Roberts-Dalton et al. labeled EVs with fluorescent molecules using this strategy and successfully analyzed the cellular dynamics of the engineered EVs by fluorescence microscopy [146]. Besides the fluorescent molecules, other targeting or reactive moieties can also be ligated to the EV surface in this manner. However, the efficiency may be lower than amino group-based modifications, as thiol groups are much less frequent than amino groups on the EV membrane surface.

Alternatively, reactive groups can be introduced to EV membranes through lipid insertion. An example of lipid insertion-assisted chemical ligation is provided in “Lipid insertion” section [138].

#### Metabolic labeling

For metabolic labeling, the reactive groups, such as azide groups, are metabolically introduced to EV membrane proteins/glycoproteins by culturing the donor cells in a medium supplied with azide-bearing amino acids or azide-containing saccharides, which allows further modification with targeting moieties through click chemistry. The method is robust and can efficiently label EV surface. However, azide-bearing supplement for large volume of medium and substrate synthesis for click chemistry will make this method costly.

Wang et al. co-cultured EV-secreting cells with l-azidohomoalanine or tetra-acetylated *N*-azidoacetyl-d-mannosamine to produce the azide-bearing EVs [147]. Both EVs allowed labeling of fluorescent molecules or biotin by click chemistry and could be employed for imaging and targeted delivery. Lee et al. adopted a similar method to obtain azide-containing EVs with tetra-acetylated *N*-azidoacetyl-d-mannosamine, which could be labeled with azadibenzylcyclooctyne-fluorescent dyes by click chemistry and used to study the uptake and distribution of EVs in cells and in vivo [148].

#### Affinity binding

For affinity binding-based modification, the targeting moieties are displayed on EV surface through linking to affinity molecules of EV membrane proteins or lipids. The affinity molecules can be peptides, proteins and aptamers. The modification can be accomplished through simple mixing and incubation. The method is less robust than the covalent bonding-based approaches, but it has no perturbation on EV membrane structure.

CD63 is specifically enriched in the exosome membrane and is used as a biomarker for exosome characterization. Its affinity peptide, CP05, can specifically bind to the second extracellular loop of CD63 as screened out by phage display [149]. Due to the convenient modification of the peptide and the strong affinity binding, CP05 represents a simple and efficient surface modification approach for exosomes [150]. Targeted delivery by CP05 modified exosomes was tested in mdx mice, with the muscle-targeting peptide, M12, and the FDA approved drug phosphorodiamidate morpholino oligomer (PMO) for treating Duchenne muscular dystrophy [149]. The engineered exosomes significantly enhanced the number of dystrophin-positive myofibers in muscles and achieved functional rescue without any detectable toxicity in mdx mice. Also, Guo et al. established a targeted delivery platform, SmartExo, which contained CP05-thioketal-mPEG on the membrane surface to anchor therapeutic peptide on the EV surface for deliver and chlorin e6 in the lumen of exosomes [151]. During circulation, SmartExo could escape phagocytosis in non-target organs due to the stealth effect of PEG, which was removed when thioketal was degraded by reactive oxygen species produced by chlorin e6 upon ultrasound irradiation. The engineered exosomes could effectively escape phagocytosis, deliver Bmp7 mRNA into omental adipose tissue (OAT), and induce OAT browning, offering a promising strategy for anti-obesity therapy. In another example, Dong et al. linked the anti-angiogenic peptide KV11 to the surface of endothelial cell-derived EVs through the affinity binding of CP05 [152]. Compared to the peptide alone, modified EVs greatly enhanced the anti-angiogenic effect.

Other than the exosomal membrane proteins, EVs inherit membrane proteins from the donor cells, which provide binding sites for modification. EVs derived from reticulocytes (RTCs) contain transferrin receptors, allowing affinity binding of transferrin proteins. Qi et al. collected RTC-derived EVs from blood under an external magnetic field using the superparamagnetic nanoparticle-transferrin conjugation and employed these EVs for the targeted delivery of doxorubicin for cancer treatment [153].

Besides the protein–protein interaction, some protein moieties could specifically bind to lipids. The affinity binding between the C1C2 domain of lactadherin and phosphatidylserine and the signal peptides derived from DAF and GPI were employed in the surface display of EVs (reviewed in “Genetic modification” section).

Also, aptamers represent a mild and specific tool for developing affinity-binding-based methods. Wan et al. developed a technique for the surface modification of exosomes based on the DNA aptamer as an exosome surface marker and DNA hybridization chain reaction initiated by an aptamer-chimeric trigger, allowing the specific labeling of exosomes with FITC [154]. The technique can be applied by replacing the FITC motif with other targeting molecules.

#### Enzymatic conjugation

Enzymatic conjugation approach is developed based on protein ligase, a kind of enzymes that can ligate proteins/peptides containing specific amino acid sequences. Surface modification are realized when applying the enzymatic ligation between EV membrane protein and targeting protein/peptide. This method has no requirement of genetic or chemical modification but creates permanent covalent modification [155].

**Fig. 5:**
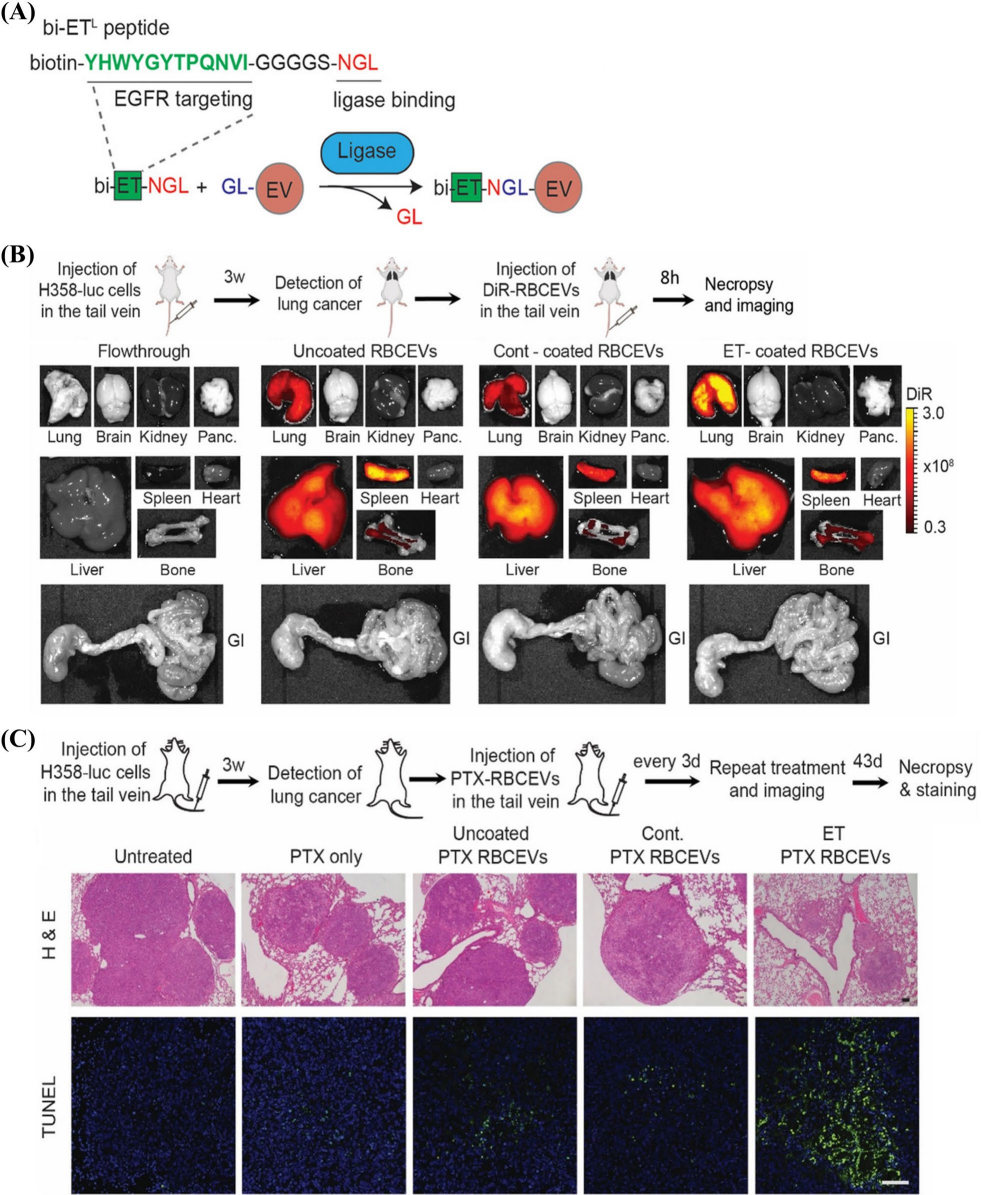
Targeted delivery of PTX by enzymatically conjugated RBCEVs for EGFR^+^ lung cancer treatment [156]. **A** Schematic diagram of the conjugation between biotin modified ET-NGL peptide (bi-EL-NGL) and GL containing EV (GL-EV) by OaAEP1 ligase. **B** Distribution of intravenously administrated uncoated RBCEVs, Cont-coated RBCEVs and ET-coated RBCEVs (DiR labeling) in different organs from EGFR^+^ lung cancer xenografted mouse model by IVIS imaging. **C** H&E staining and TUNEL assay of lung sections from EGFR^+^ lung cancer xenografted mouse model with the administration of PTX, PTX delivered by uncoated RBCEVs, PTX delivered by Cont-coated RBCEVs, and PTX delivered by ET-coated RBCEVs (TUNEL, green; Cell nucleus, blue; Scale bar = 100 μm) (Reproduced under the terms of the CC-BY 4.0. Copyright 2020, The Authors, published by Wiley Periodicals, LLC on behalf of the International Society for Extracellular Vesicles)

Pham et al. employed Sortase A and OaAEP1protein ligases to covalently modify EVs (Fig. 5) [156]. The modified copies per vesicle vary depending on specific EV types as a result of the varied amounts of enzyme-recognition motif on membrane surface. For red blood cell-derived EVs (RBCEVs), OaAEP1 ligase could ligate about 380 copies of the target peptide on vesicle, guaranteeing the targeting efficiency of the modified EVs. To target EGFR-positive cells, ET peptide, the EGFR-targeting peptide was introduced to the surface of red blood cell-derived EVs by the ligation between biotin modified ET-NGL peptide and GL containing EV, in which C-terminal NGL of the peptide and N-terminal GL on EV surface were recognition motifs for OaAEP1 ligase (Fig. 5A). ET-ligated/coated RBCEVs showed targeted delivery of PTX and significantly increased drug efficacy in xenografted mouse model of EGFR^+^ lung cancer (Fig. 5B, C).

### Membrane fusion with liposomes

The fusion of lipid double membrane is a key process in normal cell biology, and it has been adapted to exosome membrane engineering. When liposomes are incubated with EVs, the targeting motif-containing liposomes spontaneously fused with the exosome membrane, displaying the functional groups on the surface of the fused vesicles.

**Fig. 6:**
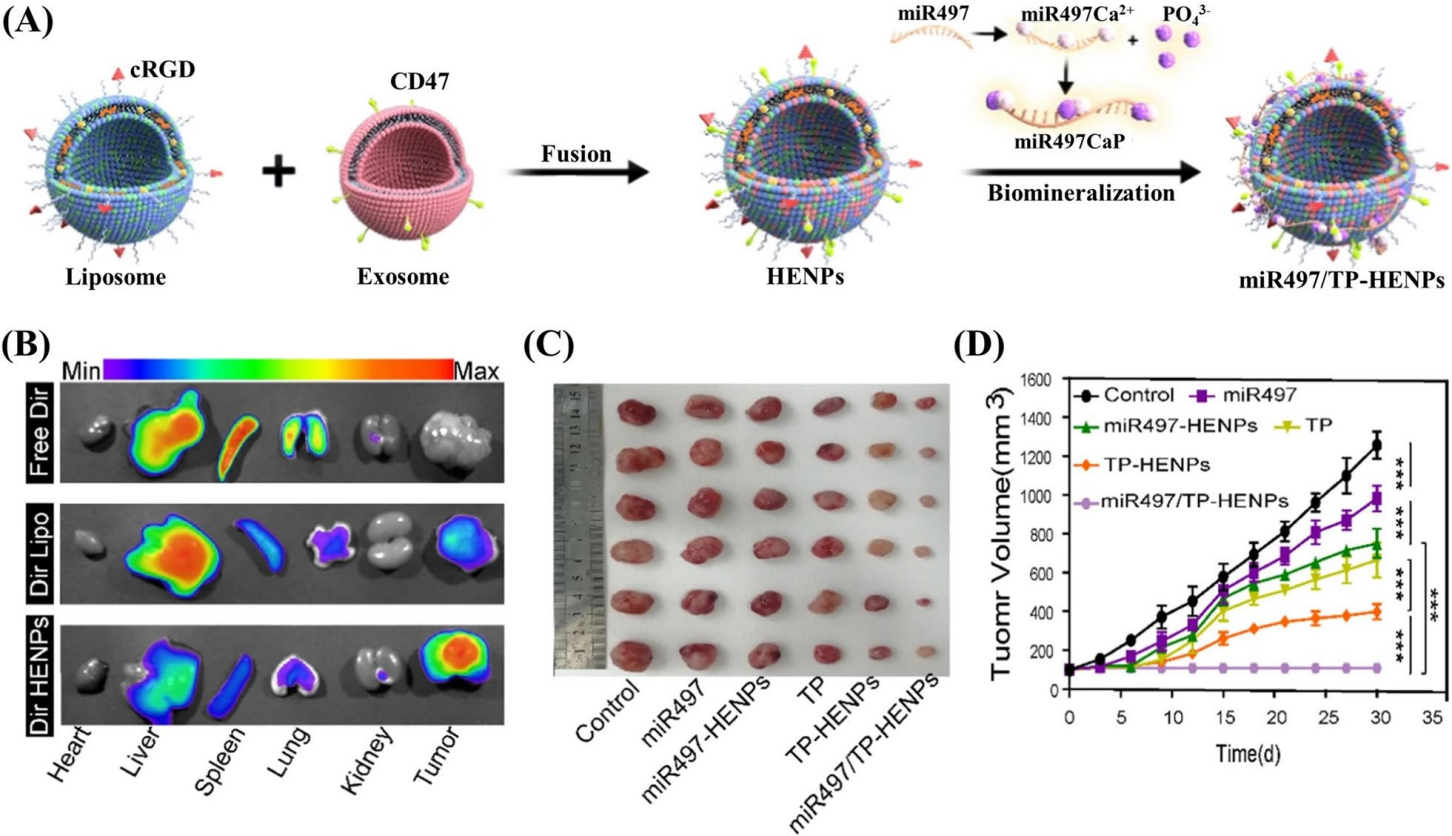
Targeted delivery of TP and miR497 by liposome-EV fused vesicels for cisplatin-resistant ovarian cancer treatment [159]. **A** Schematic diagram of the production of miR497/TP HENPs by membrane fusing between RGD-modified liposome and CD47-bearing EVs, and biomineraliazation for the encapsulation of miR497. **B** Distribution of intravenously administrated free Dir dye, Dir-labeled liposome and Dir-labeled HENPs in different organs and tumor tissue from the SKOV3-CDDP xenografted mice by the in vivo imaging apparatus. **C** The dissected tumor tissue and the tumor growth record curves of SKOV3-CDDP xenografted mice after the intravenous administration of miR497, miR497-HENPs, TP, TP-HENPs, and miR497/TP-HENPs (^***^P < 0.001 using two-way or one-way ANOVA for independent *t* test analysis by GraphPad Prism software 8.0) (Reproduced under the terms of the CC-BY 4.0. Copyright 2022, The Authors, published by BioMed Central on behalf of the Journal of Nanobiotechnology)

Li et al*.* developed a hybrid nanocarrier, HENPs, by membrane fusion of RGD-modified liposome and CD47-bearing EVs, to specifically deliver triptolide (TP) and miR497 to tumor cells for the treatment of cisplatin-resistant ovarian cancer (Fig. 6A) [159]. RGD endowed HENPs with tumor-targeting capacity, while CD47 helped to evade phagocytosis from the mononuclear phagocyte system (MPS). HENPs showed enrichment in subcutaneous SKOV3-CDDP tumors in xenograft mice, while liposomes were more trapped in liver by MPS (Fig. 6D). Moreover, miR497/TP HENPs showed great anti-tumor effect in mice bearing subcutaneous SKOV3-CDDP tumors (Fig. 6E). The hybrid nanocarrier exerted great delivery capacity for both miRNA and small molecules, emerging as a powerful tool for cancer therapy.

Besides targeting motif, liposome can introduce thermosensitive element into the fused nanoparticle for targeted therapy. Thus, hybrid nanoparticles loaded with photothermal agents can achieve excellent photothermal therapy under laser irradiation after intravenous injection. Cheng et al. fused thermosensitive liposome with CD47-overexpressing exosomes to evade phagocytosis from MPS [160]. This ideal temperature-responsive and near-infrared laser-controlled drug release system combined photothermal therapy with immunotherapy to provide effective targeted treatment for cancer. Lv et al. employed similar strategy to produce genetically engineered exosomes-thermosensitive liposomes hybrid nanoparticles (gETL NPs) for metastatic peritoneal carcinoma treatment [161]. gETL NPs were fused by thermosensitive liposome and CD47-expressing EVs, which could efficiently deliver granulocyte–macrophage colony-stimulating factor and docetaxel to tumor and release them under hyperthermic intraperitoneal chemotherapy (HIPEC). The combination of HIPEC and gETL NPs-assisted delivery of chemoimmunotherapy showed promising in vivo effects.

### Other EV membrane modifying approaches

**Fig. 7:**
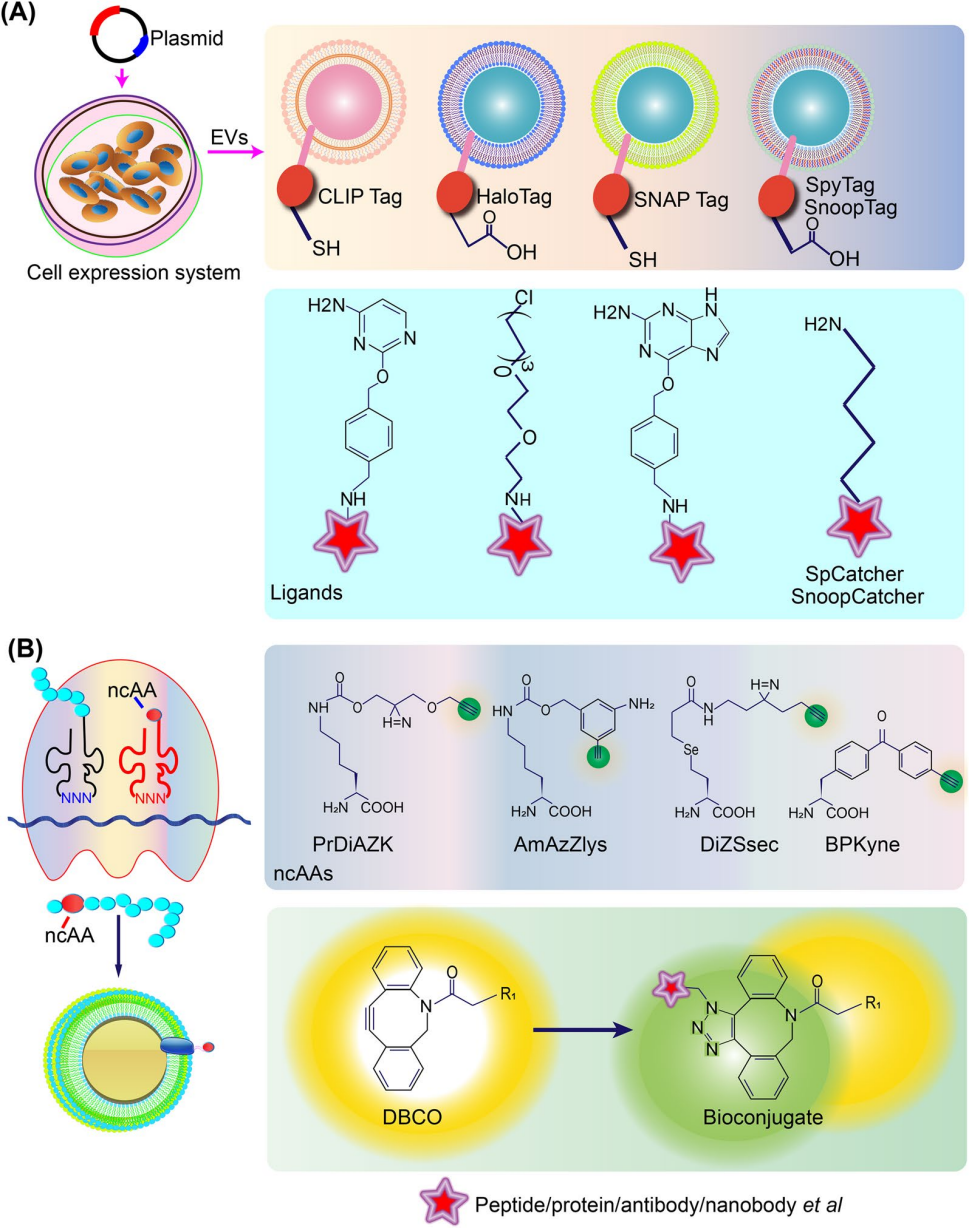
Proposed model of chemical biology approaches for surface engineering of EVs. **A** The exosomal membrane proteins fused with HaloTag, SNAP-tag, CLIP-tag, ybbR tag, SpyTag, and SnoopTag are expressed in eukaryotic cells. The specific ligands for each tag consisting of ligands conjugated with targeting moieties can be covalently immobilized on the exosome membrane with surface chemical reaction. **B** Incorporating non-canonical amino acids into EV membrane proteins can promote the azide-alkyne cycloaddition. Then an alkynyl-bearing protein is conjugated to an azide-labeled targeting moiety such as peptide/protein/antibody/nanobody etc.

EV membrane proteins can be further site-specifically modified by several strategies: enzymatic ligation using ybbR tag/CoA/SFP [162]; labeling reaction between CLIP-tag and benzylcytosine derivatives [163]; covalent bond formation between HaloTag and chloroalkane derivatives [164]; labeling reaction between SNAP-tag and *O*(6)-benzylguanine derivatives [165]; spontaneous isopeptide bond formation using the SpyTag/SpyCatcher or SnoopTag/Snoop catcher system [166, 167] (Fig. 7A). Genetic code expansion is another powerful tool for developing exosomal membrane modifications [168]. This method allows site-specific conjugation of non-canonical amino acids, including PrDiAZK, AmAzZlys, DiZSsec and BPKyne, with reactive groups, such as copper-catalyzed alkyne-azide cycloaddition (CuAAC), conjugating alkynyl-bearing EV proteins to azido-linked targeting groups (Fig. 7B).

## Conclusions and perspectives

**Fig. 8:**
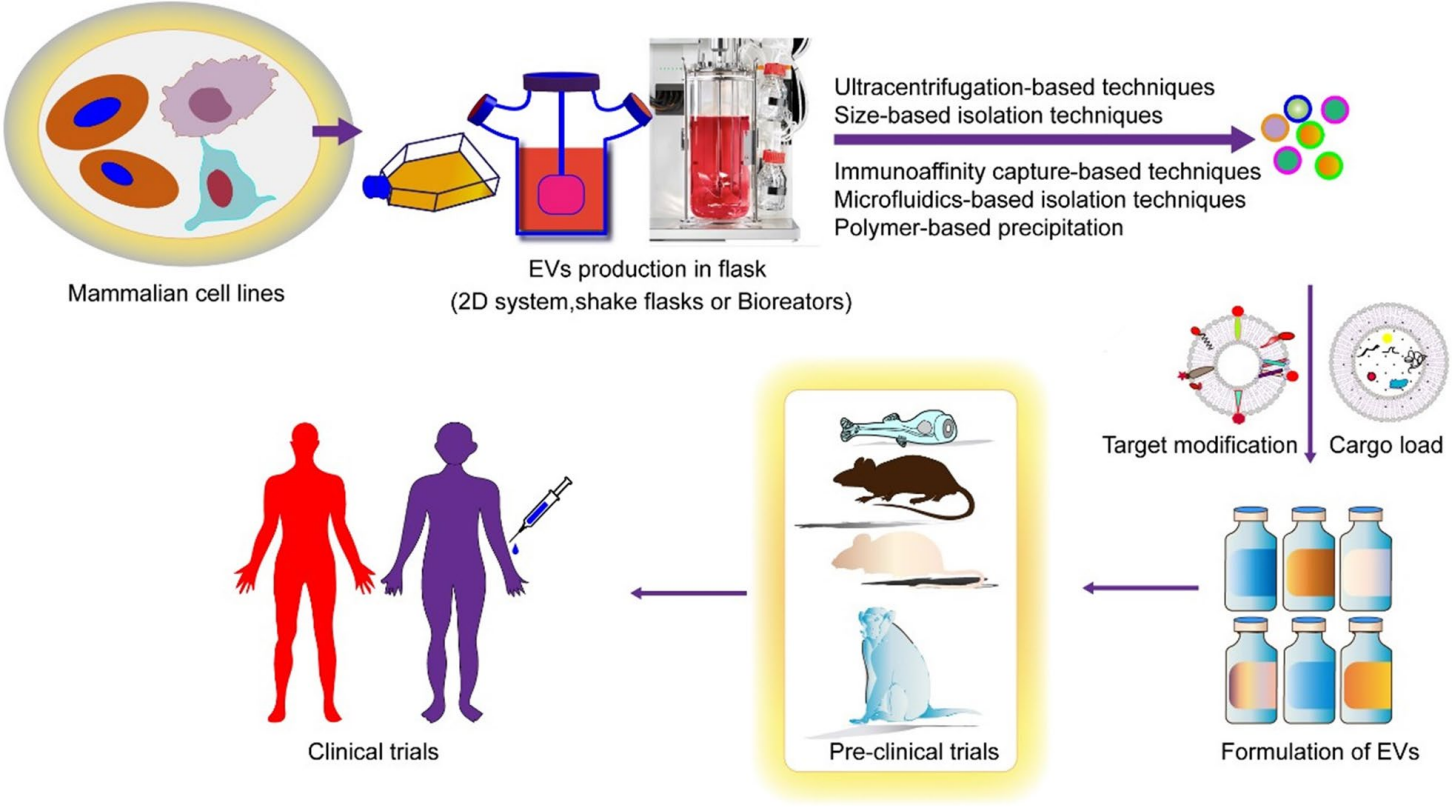
EVs applications in biomedicine

EVs provide great potential in drug delivery and disease treatment, and may become next-generation drug delivery vehicles in the future (Fig. 8). As an emerging drug carrier, EVs still face many challenges for clinical application. The low yield and insufficient purity are the main reasons that impede the wide-ranging application of EVs in clinical practice [169, 170]. Although EVs have been successfully designed to target several receptors for targeted therapy, many problems remain to be solved for EV-based drug delivery, such as low targeting efficiency. Many approaches, including genetic engineering, hydrophobic insertion, chemical modification, liposome fusion, metabolic engineering, and enzymatic remodeling are being developed to improve the targeting efficiency of EVs.

Genetically modified cells to direct the display of specific target elements on the exosomal membrane surface is a general strategy [116, 122, 126, 171]. But these methods are even less suitable for difficult-to-transfect cell types, including stem cells and red blood cells [117]. Non-genetic approaches include lipid-, glycan-, and protein-based modifications to circumvent the risks associated with genetic modification [82, 136, 146, 147, 149, 156]. These membrane surface engineering techniques have demonstrated remarkable preclinical results in tissue engineering, targeted therapy, and cellular immunotherapy. To that end, efforts are underway to make these systems more stable, general, innocuous, and reversible—features that may help overcome adverse events. In the future, EVs are expected to become a critical drug delivery carrier.

## Data Availability

Not applicable.

## Abbreviations

EVs: Extracellular vesicles
MSC: Mesenchymal stem cell
MVBs: Multivesicular bodies
ILVs: Intraluminal vesicles
ESCRT: Endosomal sorting complex required for transport
ALIX: ALG-2-interacting protein X
VPS4A/B: Vacuolar protein sorting-associated protein 4A/B
TSAP6: Tumor-suppressor-activated pathway 6
EGFR: Epidermal growth factor receptor
EGF: Epidermal growth factor
GPC-1: Glypican-1
PTX: Paclitaxel
CAR: Chimeric antigen receptor
rAAV: Recombinant adeno-associated viruses
iCasps: Inducible *caspase 9*
MDD: Major depressive disorder
IVIS: In vivo imaging system
SPT: Sucrose preference test
FST: Forced swim test
TST: Tail suspension test
OFT: Open field test
CUS: Chronic unpredictable stress
PDGFR: Platelet-derived growth factor receptor
CXCR4: Chemokine receptor 4
SMART-Exos: Synthetic multivalent antibodies retargeted exosomes
PD-1: Programmed death-1
OX40L: OX40 ligand
GEMINI-Exos: Genetically engineered multifunctional immune-modulating exosomes (GEMINI-Exos)
PTGFRN: Prostaglandin F2 receptor negative regulator
PSA: Prostate-specific antigen
PAP: Prostatic acid phosphatase
DAF: Decay-accelerating factor
GPI: Glycosylphosphatidylinositol
DSPE-PEG: 1,2-Distearoyl-*sn*-glycero-3-phosphoethanolamine-Poly(ethylene glycol)
GBM: Glioblastoma
DSPE-PEG-Mal: Meleimide (Mal)-terminated DSPE-PEG
STAT3: Signal transducers and activators of transcription 3
DOPE-PEG: 1,2-Dioleoyl-*sn*-glycero-3-phosphoethanolamine-Poly(ethylene glycol)
SPION: Superparamagnetic iron oxide nanoparticles
CuAAC: Copper-catalyzed alkyne-azide cycloaddition
MCAO: Middle cerebral artery occlusion
BBB: Blood brain barrier
PMO: Phosphorodiamidate morpholino oligomer
OAT: Omental adipose tissue
RTCs: Reticulocytes
RBCEVs: Red blood cell-derived EVs
TP: Triptolide
MPS: Mononuclear phagocyte system
gETLNPs: Engineered exosomes-thermosensitive liposomes hybrid nanoparticles
HIPEC: Hyperthermic intraperitoneal chemotherapy
